# Primary biliary cirrhosis presenting with ascites and a hepatic hydrothorax: a case report

**DOI:** 10.4076/1752-1947-3-7371

**Published:** 2009-07-14

**Authors:** Thomas George Wojcikiewicz, Sachin Gupta

**Affiliations:** 1 Department of Gastroenterology, Princess Alexandra Hospital, Harlow, Essex, UK

## Abstract

**Introduction:**

We report an unusual presentation of primary biliary cirrhosis.

**Case presentation:**

We present the case of a 66-year-old white British woman who presented to the Accident and Emergency department with ascites and unilateral pleural effusion (hepatic hydrothorax). Following a liver biopsy, the diagnosis of primary biliary cirrhosis was made.

**Conclusion:**

It is important to consider the diagnosis of hepatic cirrhosis when presented with a unilateral pleural effusion in both the presence and absence of ascites.

## Introduction

Primary biliary cirrhosis (PBC) occurs when interlobular bile ducts are damaged by chronic granulomatous inflammation. This causes progressive cholestasis, cirrhosis and portal hypertension.

Often asymptomatic, diagnosis usually comes about following the discovery of a raised alkaline phosphatase on routine liver function tests (LFTs). Symptoms include lethargy and pruritis. Clinical signs include jaundice, skin pigmentation, xanthelasma, xanthomata, hepatomegaly and splenomegaly.

Complications of the disease include osteoporosis, malabsorption of fat soluble vitamins, portal hypertension, ascites, variceal haemorrhage and hepatic encephalopathy.

Hepatic hydrothorax is a pleural effusion occurring in patients with cirrhosis and without cardiac or pulmonary disease. It is a relatively uncommon complication and occurs when ascitic fluid moves from the peritoneum into the pleural space. The fluid movement occurs through defects in the diaphragm.

## Case presentation

A 66-year-old woman presented to the Accident and Emergency department following three episodes of melaena and one episode of haematemesis. On further questioning, she also reported a 4-month history of worsening shortness of breath, initially when using stairs and more recently, after walking only a few yards. In addition to this, she admitted to abdominal distension over a number of months and weight loss of approximately 2 stone (approximately 13 kg) over the period of a year.

She had no significant previous medical history and was not taking any regular medications. She was an ex-smoker, only drank occasional alcohol and was fully independent but recently had to rely on her husband for help with domestic chores due to her shortness of breath.

On examination, she was noted to be tachypnoeic, tachycardic but with normal oxygen saturation and blood pressure. A cardiovascular examination was normal. Breath sounds were reduced in the entire right lung field, while the left lung was normal. Her abdomen was distended with tenderness on deep palpation in the left iliac fossa. There was no organomegaly. Shifting dullness was present and bowel sounds were normal. A digital rectal examination demonstrated a prolapsed rectum with fresh blood and dark stool on the finger tip. She was alert and fully orientated.

Chest X-ray demonstrated a large right pleural effusion, with an almost complete white-out.

Admission bloods demonstrated a normocytic anaemia: Hb 8.4 g/dL, low ferritin, normal vitamin B12 and folate, albumin 31 g/L, alkaline phosphatase 170 IU/L, ALT 32 IU/L, corrected calcium 2.28 mmol/L.

Whilst hospitalised, she was incontinent with a large amount of melaena which caused her to become haemodynamically unstable. She was fluid resuscitated and a chest drain was inserted.

Given her history and presentation, a diagnosis of malignancy, possibly disseminated, was suspected. Samples of pleural and ascitic fluid both demonstrated transudates with low LD/LDH. Cytology was normal with no malignant cells seen. An oesophagogastroduodenoscopy (OGD) demonstrated grade 4 oesophagitis and gastritis. A subsequent colonoscopy was normal. Ultrasound of her abdomen reported normal liver and kidneys but splenomegaly. CA 125 was found to be raised. Consequently, an ultrasound scan of her pelvis showed no gynaecological malignancy. A barium follow-through was arranged. This was normal but with some jejunisation of the ileum.

The derangement of the LFTs led to a full liver screen. An autoimmune profile demonstrated the presence of antimitochondrial M2 antibodies, antinuclear antibody (ANA) positive, and extractable nuclear antigen (ENA) positive. IgM was also elevated.

A liver biopsy gave the diagnosis of primary biliary cirrhosis.

## Discussion

In 1876, Hanot first described the symptoms of PBC: jaundice, pruritis, fatigue, hepatomegaly and splenomegaly. It was initially called 'Hanot's cirrhosis'. It was Dauphinee and Sinclair who first used the term 'primary biliary cirrhosis'. They recognised that in the latter stages of the disease, there are the complications of cirrhosis and portal hypertension; oesophageal varices and fluid retention, namely, ascites [1]. Ascites is a result of portal hypertension which leads to fluid accumulation within the abdomen. This usually causes a transudate. Exudative ascites is usually a result of malignancy.

Ascites itself can be classified in grades 1-3. Grade 1 ascites is mild and only detectable by ultrasound. Grade 2 ascites is detectable clinically with moderate symmetrical distension of the abdomen. Grade 3 ascites is large or gross ascites; there is marked abdominal distension [2].

A hepatic hydrothorax is defined as a pleural effusion in a patient with cirrhosis of the liver and no cardiopulmonary disease. The estimated prevalence of this often debilitating complication in patients with liver cirrhosis is 4% to 10% [3]-[6]. The hepatic hydrothorax usually develops on the right side (85%), followed by the left (13%), with the remainder being bilateral (2%) although some literature has quoted 16% being on the left side and 16% bilateral [7,8].

The pleural effusion is derived from the ascites due to negative pressure in the pleural space - a pressure gradient develops between the intraperitoneal and intrapleural spaces. The ascites moves via defects in the diaphragm. The defects are usually small and in the tendinous portion of the diaphragm, often <1 cm in size. Sometimes the defects are macroscopic.

Studies have shown that the flow from the peritoneum into the pleural cavity is unidirectional. Work by Rubinstein et al. demonstrated this in two cases of hepatic hydrothorax by injecting the radioisotope 99Tc-sulphur colloid into the peritoneal space. They showed that there was one-way transdiaphragmatic flow of fluid from the peritoneal to the pleural cavities. However, radioisotope injected into the peritoneal cavity of five patients with pleural effusions secondary to pulmonary or cardiac disease failed to traverse the diaphragm and localized in the pleural space [9].

Hydrothoraces are often progressive presenting with shortness of breath, cough, and chest discomfort. They can be relatively asymptomatic until large. Ascites is not always present. The composition of hydrothoraces is transudative in nature and similar to the ascitic fluid with few cells, predominantly lymphocytes and mesothelial cells. The protein content is often slightly greater than that of ascitic fluid [10].

The management of hepatic hydrothoraces is based around sodium restriction and diuretics; the combination of furosemide and spironolactone has been found to be very effective [11]. Some patients do not respond to this first-line treatment even when diuretics are at their maximal doses. For these patients, treatment options include repeated thoracentesis, transjugular intrahepatic portosystemic shunt (TIPS), pleurodesis and repair of the diaphragmatic defects.

## Conclusion

When presented with a unilateral pleural effusion, either in the presence or absence of abdominal ascites, it is important to consider hepatic cirrhosis as a possible cause.

## Abbreviations

ALT: alanine aminotransferase; ANA: antinuclear antibody; ENA: extractable nuclear antigens; LFTs: liver function tests; OGD: oesophagogastroduodenoscopy; PBC: primary biliary cirrhosis; TIPS: transjugular intrahepatic portosystemic shunt.

## Consent

Written informed consent was obtained from the patient for publication of this case report. A copy of the written consent is available for review by the Editor-in-Chief of this journal.

## Competing interests

The authors declare that they have no competing interests.

## Authors' contributions

TW collected the information and carried out the research. He was the main writer of the manuscript. SG advised, read and approved the final version.
